# Effect of Water Chemistry, Land Use Patterns, and Geographic Distances on the Spatial Distribution of Bacterioplankton Communities in an Anthropogenically Disturbed Riverine Ecosystem

**DOI:** 10.3389/fmicb.2021.633993

**Published:** 2021-05-06

**Authors:** Jun Zhao, Wang Peng, Mingjun Ding, Minghua Nie, Gaoxiang Huang

**Affiliations:** ^1^School of Geography and Environment, Jiangxi Normal University, Nanchang, China; ^2^Key Laboratory of Poyang Lake Wetland and Watershed Research, Ministry of Education, Jiangxi Normal University, Nanchang, China

**Keywords:** bacterioplankton community, mass effects, species sorting, riverine ecosystem, variation partitioning

## Abstract

The spatial distribution of bacterioplankton communities in rivers is driven by multiple environmental factors, including local and regional factors. Local environmental condition is associated with effect of river water chemistry (through species sorting); ecological process in region is associated with effects of land use and geography. Here, we investigated variation in bacterioplankton communities (free-living, between 0.22 and 5 μm) in an anthropogenically disturbed river using high-throughput DNA sequencing of community 16S rRNA genes in order to investigate the importance of water chemistry, land use patterns, and geographic distance. Among environmental factors, sulfate (SO_4_^2–^), manganese (Mn), and iron (Fe) concentrations were the water chemistry parameters that best explained bacterioplankton community variation. In addition, forest and freshwater areas were the land use patterns that best explained bacterioplankton community variation. Furthermore, cumulative dendritic distance was the geographic distance parameter that best explained bacterial community variation. Variation partitioning analysis revealed that water chemistry, land use patterns, and geographic distances strongly shaped bacterioplankton communities. In particular, the direct influence of land use was prominent, which alone contributed to the highest proportion of variation (26.2% in wet season communities and 36.5% in dry season communities). These results suggest that the mechanisms of species sorting and mass effects together control bacterioplankton communities, although mass effects exhibited higher contributions to community variation than species sorting. Given the importance of allochthonous bacteria input from various land use activities (i.e., mass effects), these results provide new insights into the environmental factors and determinant mechanisms that shape riverine ecosystem communities.

## Introduction

Bacterioplankton compositions reflect environmental changes in riverine ecosystems due to the sensitivity of their responses to environmental conditions (Marshall et al., 2008). The biotic community responds to changes in environmental conditions from upstream to downstream, as defined by the River Continuum Concept (Vannote et al., 1980); and responds to lateral exchange from the main river channel/lake to the connected floodplain, as defined by Flood pulse concept (Junk et al., 1989). Terrestrial environments serve as critical sources of the microbial community for river surface waters (Crump et al., 2012), and the bacterioplankton community can exhibit certain spatial distributions along a river’s length. Bacterial communities passively disperse in rivers and exhibit certain spatial distributions along water flow paths extending from the river bank to the channel in the transverse dimension or from upstream to downstream in the longitudinal dimension (Crump et al., 2012). In river ecosystems, changes in river water quality and flow hydraulics/hydrology act together with changes that affect bacterial dispersal and alter spatial patterns of community assembly (Philippe et al., 2020).

Numerous studies have indicated that bacterioplankton communities are affected by many environmental factors (Niño-García et al., 2016b; Huang and Huang, 2019; Wang et al., 2019) including water chemistry, land use types, and geographic distances. Water chemistry components (e.g., pH, nutrient contents, and heavy metal concentrations) directly affect the habitats of bacterioplankton communities and alter their spatial variation. In addition, anthropogenically disturbed riverine ecosystems that are reflected in land use urbanization, industrialization, and agriculturalization affect the spatial distributions of bacterioplankton communities. Furthermore, bacterioplankton communities are affected by water chemistry due to the input of allochthonous nutrients or heavy metals (Vishnivetskaya et al., 2011; Staley et al., 2014) in addition to the direct effects input of allochthonous bacteria to the river (Staley et al., 2015; Wang et al., 2019). Moreover, land use patterns alter the characteristics of river networks including through placement of reservoirs. Lake/reservoir tends to have unique water chemistry (e.g., dissolved oxygen and nutrient levels) and hydraulic/hydrologic characteristics (e.g., flow rates and water residence time), and the shift in compositions can occur as bacterioplankton communities transit the lake/reservoir (Crump et al., 2007; Stewart et al., 2011; Adams et al., 2014). Finally, river network characteristics affect bacterial community interactions (e.g., through predation and growth competition) and drive the spatial distributions of bacterial communities (Savio et al., 2015). A better understanding of the determinant factors that drive riverine microbial communities is crucial for understanding the underlying mechanisms affecting riverine ecosystem functioning (Mo et al., 2018). However, interactive effects among the environmental factors that affect bacterial communities remain unclear in these ecosystems. Research on these determinant factors has driven focus on the determinant mechanisms (Adams et al., 2014; Niño-García et al., 2016a; Wang et al., 2019).

Variation in riverine bacterial communities can be parsed into two determinant mechanisms: species sorting and mass effects. Species sorting emphasizes spatial niche separation, wherein relatively low levels of dispersal allow communities to respond to local conditions, and communities are directly affected by changes in environmental habitats (Leibold and Wilbur, 1992; Marmen et al., 2020). Mass effects allow inferior competitors to persist in the community and occur when relatively high levels of dispersal occur from other habitats, leading to bacterioplankton communities being directly affected by inputs of allochthonous bacteria (Urban, 2004). We hypothesized that water chemistry exerts species sorting effects on bacterioplankton communities due to predation or competition more strongly affecting some species in particular environments (Leibold et al., 2004; Crump et al., 2012; Fortunato et al., 2012; Adams et al., 2014). In addition, land use patterns and geographic distance also directly affect water chemistry due to input of allochthonous nutrients or heavy metals along with variation in water residence times. Moreover, the mass effects exerted by land use patterns and geographic distances on bacterioplankton communities could be due to allochthonous inputs from a variety of different land types at specific locations along river networks. Consequently, we further hypothesized that species sorting and mass effects work together to drive the spatial distribution of bacterioplankton communities (Supplementary Figure 1). Previous studies have attempted to understand the determinant mechanisms underlying bacterioplankton community variation by investigating various environmental factors (Liao et al., 2016; Hu et al., 2018; Wang et al., 2019), while the relative importance of these determinant mechanisms has remained unclear.

The Yuan River is a tributary of the Ganjiang River, which is the largest tributary of Poyang Lake, China. Yuan River has diverse riparian habitats from upstream to downstream, like forest, mountains, cities, and even a reservoir along streams in the middle reaches. Previous studies have determined that the Yuan River contains abundant organic pollutants (Lei et al., 2019). Moreover, the concentrations of ammonia nitrogen, suspended solids, and petroleum are high in the river after it passes through industrial city areas (Li and Liu, 2015). Lei et al. (2019) observed that land use patterns were associated with the spatial distribution of water chemistry variation in this study area. Furthermore, Yang et al. (2012) and Lei et al. (2019) investigated the relationships between river water chemistry and phytoplankton/benthic animal communities in this study area, respectively. However, a limited understanding exists for the role of environmental factors in shaping bacterioplankton communities, and the determinant mechanisms that shape bacterioplankton communities of rivers are unknown. Consequently, the objectives of our study were to (1) understand how water chemistry, land use patterns, and geographic distances differentially shape spatial variation in bacterioplankton communities and (2) identify the determinant mechanisms (i.e., species sorting or mass effects) that control bacterioplankton community composition. The results from this study provide new insights into the determinant mechanisms underlying bacterioplankton variation in riverine ecosystems and know more detail about bacterioplankton response to reservoir construction.

## Materials and Methods

### Study Sites

**FIGURE 1:**
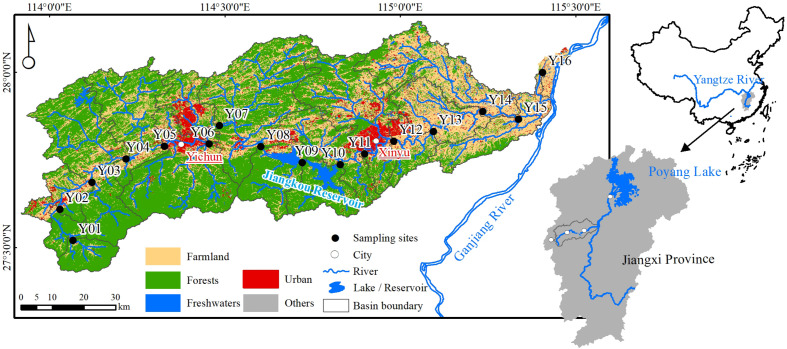
Map of the Yuan River and sampling sites. The cities of Pingxiang, Yichun, and Xinyu are located upstream and downstream in the Yuan River basin.

The Yuan River is located in the lower reaches of the Ganjiang River, which is a major tributary of the Yangtze River and directly feeds Poyang Lake (Figure 1). The length of the main river channel is approximately 297 km, and its basin area is 6,262 km^2^. The cities of Pingxiang, Yichun, and Xinyu are located in the Yuan River basin. The climate of the region is influenced by subtropical monsoon patterns, with a wet season between April and September and a dry season from October to March.

The Yuan River passes through various landforms including mountains, foothills, and plains (Supplementary Figure 2). The Pingxiang coal mines are famous in China and located in the upper reaches. In the middle reaches, the Jiangkou Reservoir (also referred to as Fairy Lake) stores 320 million m^3^ of water and generates 9,160 million kwh every year. Mechanical, electrical, textile, chemical, and metal industries (e.g., iron and steel) are all prevalent in the Jiangxi Province and are located in Xinyu City. Farmland is mostly present in the downstream areas, and their primary crop is rice. Here, forest (accounting for 52.64%) and farmland (35.10%) were the main land use types (Figure 1 and Supplementary Table 1).

### Water Sampling and Measurement of Environmental Factors

In total, 16 sites were sampled (Figure 1 and Supplementary Table 1). River water samples were collected in the wet season (August 2018) and in the dry season (January 2019) from five upper reach sites (Y01–Y05), six sites in the middle reaches (Y06–Y11), and five sites in the lower reaches (Y12–Y16). At each sampling site, surface water samples (approximately 0.5 m below the surface) were collected in triplicate at intervals of 5 m with a portable plexiglass water sampler (Beijing Pulite Instrument Co., Ltd., China) and then pooled together into a single sample.

Dissolved oxygen (DO) and pH were measured *in situ* using an HI 98360 probe (Hanna Instruments Ltd., Italy) that was calibrated before each measurement. Samples were processed within 4 h of collection and then subdivided for chemical and molecular analyses. Four water samples were collected in separate 100-ml polyethylene bottles at each site: (1) one for dissolved organic carbon (DOC) analysis, (2) one for ammonium (NH_4_^+^-N), nitrate (NO_3_^–^-N), chloride (Cl^–^), and sulfate (SO_4_^2–^) measurements, (3) one for total phosphorus (TP) analysis, and (4) one for heavy metal measurements. Water samples were filtered through a 0.45-μm Durapore membrane filter (diameter 25 mm; Xinya, China) for all chemical analyses, except TP. Water samples were then acidified to pH 2 with ultra-purified HNO_3_ for heavy metal analysis. DOC was measured with a total organic carbon auto-analyzer (Shimadzu TOC-L CPH, Japan). NH_4_^+^-N and TP were measured using a Smartchem 200 Discrete Analyzer (Brookfield, United States). NO_3_^–^-N, Cl^–^, and SO_4_^2–^ were measured using ion chromatography (Dionex ISC2100, United States). The concentrations of manganese (Mn), iron (Fe), zinc (Zn), and lead (Pb) were measured by inductively coupled plasma mass spectrometry (ICP-MS, Thermo X series II, NE, United States).

Digital elevation model (DEM) data (at a 30 m resolution) were used to delineate basin boundaries. Sub-basin classifications at each sampling site ranged from a single sampling site that encompassed the sub-basin area to the inclusion of adjacent upper sample sites to reflect the inputs of allochthonous bacteria due to the fast population growth and replacement rates of bacterial communities.

Landsat 8 satellite imagery 2017 was used to generate a land-cover classification at a 30 m resolution. Land use pattern images were obtained from the Geospatial Data Cloud^1^. Images were then binned into five classes: farmlands, forests, freshwaters, urban areas, and others. ArcGIS v 10.3 was used to delineate basin boundaries and calculate land use proportions.

Four geographic distance parameters including river length, catchment area, cumulative dendritic distance, and mean dendritic stream length were calculated (Supplementary Table 1). River length corresponds to the length of the mainstream of the river. Most anthropogenic land use types (e.g., cities, towns, and industries) were concentrated along the mainstream. Consequently, river length reflected greater human activity than the other three geographic distance parameters. Catchment area corresponded to the basin area for each site and reflects runoff volume. Cumulative dendritic distance is a measurement – cumulative length of stream paths that from the river networks upstream of a sampling site is calculated. Mean dendritic stream length was the ratio of the cumulative dendritic stream length to the number of paths from all of the springs to the sampling site. All geographic measures were calculated using ArcGIS v.10.3.

### DNA Extraction and Illumina DNA Sequencing

Samples were pre-filtered through a 5-μm Durapore membrane filter (diameter 25 mm; Xinya, China) to remove particulates and algal biomass, followed by filtering through a 0.22-μm Durapore membrane filter (diameter 25 mm; Xinya, China) to collect microbial cells. Each water sample was simultaneously filtered through several filters to reduce filtering time. Filters from each sample were mixed and stored at −80°C for subsequent DNA extraction.

Total DNA was extracted from the water samples using the E.Z.N.A.^®^ Soil DNA Kit (Omega Bio-tek, United States). The bacterial V4–V5 hypervariable regions of 16S rRNA genes were amplified using the forward primer 338F (5′-ACTCCTACGGGAGGCAGCA-3′) and reverse primer 806R (5′-GGACTACHVGGGTWTCTAAT-3′) (Castrillo et al., 2017). PCRs were conducted using the following PCR cycling parameters: initial denaturation for 2 min at 95°C, followed by 25 cycles of 30 s at 95°C, annealing for 30 s at 55°C, and elongation for 30 s at 72°C, all followed by a final elongation step for 5 min at 72°C. Gel electrophoresis on 2% agarose gels was used to ensure to evaluate successful PCR amplification. Triplicate PCR amplicon products were pooled for each sample, purified using an AxyPrep DNA gel extraction kit (Axygen, United States), and quantified using the QuantiFluor^TM^ – ST system (Promega, United States). DNA sequencing was conducted on the Illumina MiSeq platform (Illumina, United States) following standard operating procedures and paired-end 2 × 250 bp sequencing chemistry. The sequencing was conducted at the Shanghai Majorbio Bio-Pharm Technology Co., Ltd., of China. Raw sequence data files were deposited in the NCBI Sequence Read Archive database (Accession number: SRP194014).

### Statistical Analyses

Bacterioplankton sequence data were quality filtered using the Quantitative Insights Into Microbial Ecology (QIIME) pipeline^2^ with the following criteria (Caporaso et al., 2010): (1) 250 bp reads were truncated at any base receiving an average quality score below 20 over a 50-bp sliding window and the subsequently truncated reads that were shorter than 50 bp were removed; (2) reads containing more than two nucleotide mismatches to primers or ambiguous bases were removed; and (3) only sequences that overlapped by >10 bp were assembled based on overlapping sequence. Reads that could not be assembled were discarded. Spurious sequence reads due to sequencing errors and chimeras were identified and removed using Usearch v.7.0^3^. The QIIME platform was then used to cluster reads into operational taxonomic units (OTUs) at the 97% nucleotide similarity level. The phylogenetic affiliation of each sequence was then evaluated using the RDP Classifier v.2.2^4^ and comparison against the Silva database (release 128)^5^. The 16S rRNA gene taxonomic classifications were identified based on a confidence threshold of 70% (Wang et al., 2007). Uneven sampling of sequence depth can affect statistical analyses, and thus the OTU table was randomly subsampled to ensure an equal number of sequences per sample, equivalent to the minimum number of sequences across all samples. The OTU distributions were then used to calculate alpha-diversity and beta-diversity metrics. α-Diversity metrics including the Shannon diversity and Chao1 richness indices were calculated using Mothur v.1.30.2^6^. In addition, β-diversity was calculated using Bray–Curtis distances among samples to evaluate between-sample community differences.

Prior to statistical analysis, all variables were checked for homogeneity and normal distributions using Shapiro–Wilk tests. Water chemistry parameters that were not normally distributed were log-transformed to achieve (or better approach) data normality. Pairwise comparisons of data among sub-classes were conducted using unpaired Wilcoxon rank-sum tests. Analysis of variance (ANOVA) was conducted to test significant differences in water chemistry parameters. A *t*-test was used to investigate significant differences of water chemistry parameters across seasons. Pearson correlation analysis was used to identify relationships between water chemical parameters and to identify collinearity using the SPSS Statistics software program v.20.0. Hierarchical clustering analysis was conducted using the “hclust” function in the R v.3.6.1 statistical software package. Non-metric multidimensional scaling (NMDS) analysis was used to investigate community compositional differences based on the Bray–Curtis distances of bacterioplankton communities using the “metaMDS” function in the “vegan” package for R. Lastly, an analysis of similarity (ANOSIM) test was used to evaluate significant differences between sample groups based on Bray–Curtis distances.

Redundancy analysis (RDA) was used to identify relationships in the distribution of phyla in the communities and environmental factors using the CANOCO 4.5 software suite (Ter Braak and Smilauer, 2002). The statistical significance of the explanatory power of the first ordination and the canonical axes together were assessed using permutation tests with 499 unrestricted Monte Carlo permutations (*p* < 0.05 was considered as the statistical significance threshold). Prior to RDA, variance inflation factors (VIFs) were computed to evaluate multicollinearity of multiple regression models for water chemical parameters. The “vif.cca” function of the “vegan” package for R was used to sequentially exclude water chemical parameters with VIFs > 3. The “bioenv” function in the “vegan” package was then used to identify subsets of environmental factors that best predicted microbial community structural differences. The Bioenv and RDA approaches were together used to identify the environmental factors that most contributed to microbial community variation. Environmental factors were selected from these analyses and then used in variance partitioning analysis (VPA) modeling to assess the individual relative contributions of water chemistry, land use, and geographic distance parameters.

To distinguish the influence of water chemistry, land use, and geographic distance parameters, VPA considering the three two-dimensional matrix tables for each parameter type subset was used to explain variation of community differences. The overall analysis was divided into eight independent components (Supplementary Figure 3). The procedure was conducted as follows: (1) water chemistry was constrained, then the species canonical ordinations were performed to obtain the fraction of variation that water chemistry explained [a + d + f + g]; (2) land use pattern parameters were constrained and the species canonical ordinations were performed to obtain the fraction of variation that land use explained [b + d + g + e]; (3) geographic distance parameters were constrained and the species canonical ordinations were performed to obtain the fraction of variation that geographic distance explained [c + g + f + e]; and (4) water chemistry, land use, and geographic distance were individually represented by [a], [b], and [c], respectively. Bacterioplankton community structure was then evaluated based on the intersection of water chemistry and land use factors as [d + g]; land use and geographic distance parameters as [g + e]; water chemistry and land use parameters as [f + g]; and the intersection of the above variances as [g]; the unexplained variation in bacterioplankton community structure was then 1-[a + b + c + d + e + f + g]. It should be noted that components of variation may be negative due to random variation in adjusted R^2^, although other factors can be present like multivariate non-linear dependence between sources of variation (Oksanen, 2012). Thus, negative components of variation would not be present in the results. VPA was conducted using the “varpart” function in the vegan package for R (Borcard et al., 1992).

## Results

After subsampling reads to 35,051 reads per sample, a total of 3,945 OTUs were identified in the communities using a 97% nucleotide sequence identity threshold. OTU numbers, sequence reads, and taxonomic diversity were higher in the dry season communities than wet season communities (Supplementary Table 2). All rarefaction curves approached the saturation plateau (Supplementary Figure 4), indicating that the number of sequence reads used to investigate the communities was adequate for recovering diversity. Furthermore, Good’s coverage estimates for the number of observed OTUs were 99.04 ± 0.48%, indicating that sufficient sequencing depth was used to evaluate community diversity. In addition, rarefaction curves for wet season samples plateaued quicker than dry season curves.

### Spatial Variability in Bacterioplankton Communities

**FIGURE 2:**
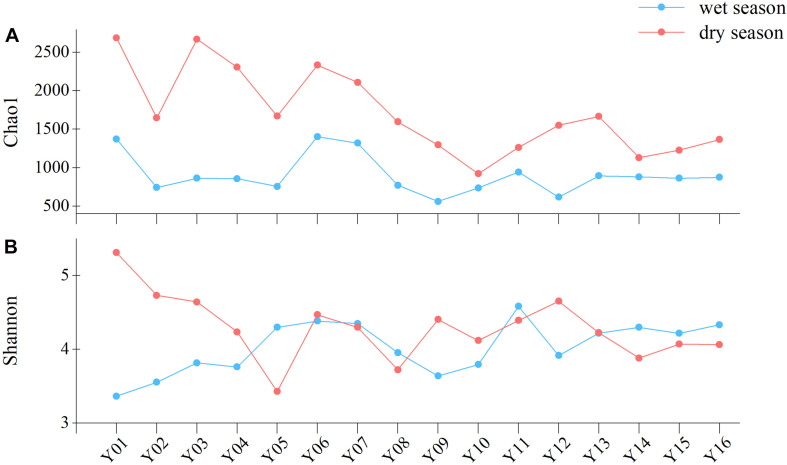
Bacterioplankton community richness and diversity in wet and dry season communities, as measured with the **(A)** Chao1 richness index and **(B)** Shannon diversity index.

Chao1 richness index values were lower in the region of Jiangkou Reservoir samples (Y09 and Y10) compared to other sample sites (Figure 2). In the dry season, both the Shannon and Chao1 index values exhibited maximum values at the Y01 site (5.31 and 2,689.03, respectively). The Chao1 richness index was significantly higher in the upper reaches than in the lower reaches (*p* = 0.022) in the dry season communities (Supplementary Figure 5). Both Shannon and Chao1 richness index value increased in the direction of river flow at site Y11 in Xinyu City, especially in the dry season.

**FIGURE 3:**
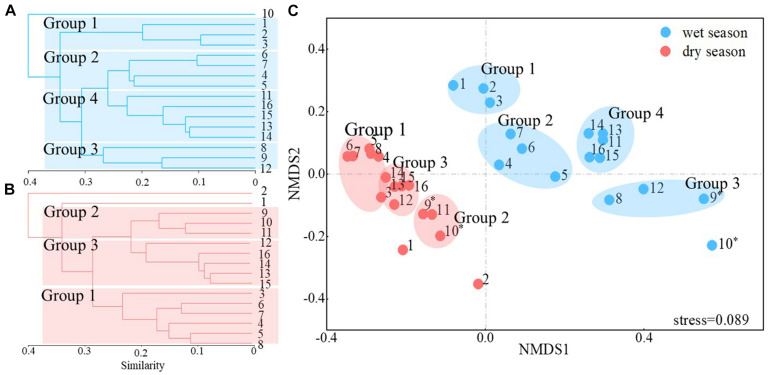
Cluster analysis and non-metric multidimensional scaling (NMDS) ordinations of Bray–Curtis distances among communities from 16 sampling sites. The numbers 1–16 correspond to the sampling sites Y01–Y16. Cluster analysis of bacterioplankton communities in the wet season **(A)** and dry season **(B)**. Based on the NMDS plots **(C)**, groups 1, 2, 3, and 4 are shown in blue and represent the five groups in the wet season. In addition, groups 1, 2, and 3 are shown in red and represent the three primary dry season groups. * indicates that the samples were collected in the region of the Jiangkou Reservoir.

**FIGURE 4:**
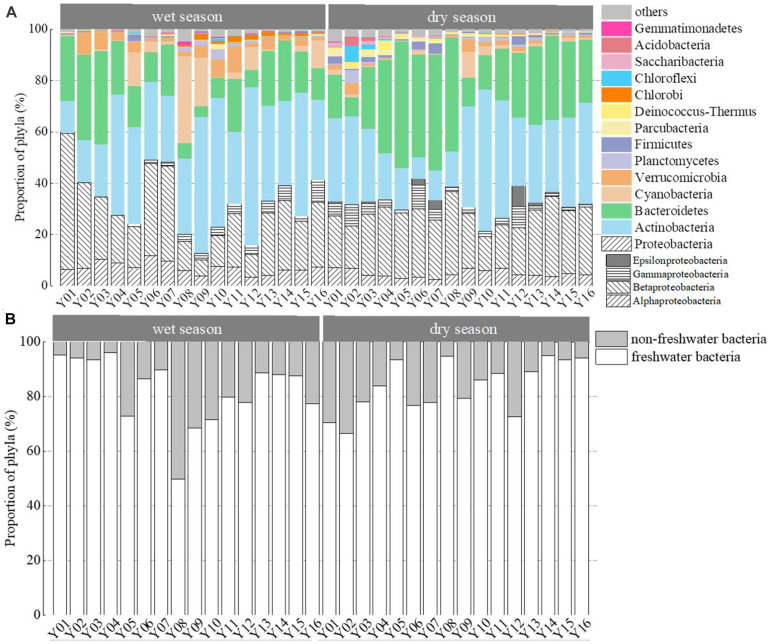
Relative abundances of bacterioplankton communities from the wet and dry seasons, arranged from upstream to downstream. Compositions are shown as **(A)** bacterioplankton phyla distributions and **(B)** freshwater and non-freshwater bacterial OTU distributions. Taxa with relative abundances <1% are grouped as “others.”

Clustering and ordination analysis clearly distinguished four groups of wet season communities and three groups of dry season bacterioplankton communities that were based on the direction of river flow (Figure 3). In addition, communities collected in the wet season clustered together, while those from the dry season clustered together. An ANOSIM test yielded a global *R* value of 0.798 at *p* < 0.001, indicating statistically significant separation of the two seasonal groups. The distribution of the dominant phyla led to distinct clustering of samples (Figure 4A). The relative abundances of Proteobacteria were significantly lower in Group 3 communities (samples Y08, Y09, and Y12) in the wet season compared to other communities (*p* < 0.05, Supplementary Table 3). In the wet season, Actinobacteria were the dominant phyla in the region of Jiangkou Reservoir communities and also in the Xinyu City communities (Y09, 52.97%; Y10, 50.36%; Y12, 61.68%). In addition, the Actinobacteria exhibited higher relative abundances in Group 3 communities (Y08, Y09, and Y12) in the wet season compared to other groups. Actinobacteria were also the dominant phylum in dry season communities in the region of Jiangkou Reservoir (Y09, 39.31%; Y10, 55.22%) and Xinyu City communities (Y11, 45.83%). The relative abundances of Actinobacteria in Group 2 dry season communities (Y09, Y10, and Y11) were significantly higher than in other communities. Bacteroidetes exhibited their lowest abundances in wet season communities in the region of Jiangkou Reservoir communities (Y09, 4.21%), but exhibited higher relative abundances in the upper reach communities. In addition, the relative abundances of Bacteroidetes in Group 1 wet season communities (Y01, Y02, and Y03) were significantly higher than in other groups, but exhibited their lowest relative abundances in dry season communities at sites from the upper reaches (Y01, 16.84%; Y02, 7.37%) and in the region of Jiangkou Reservoir sites (Y09, 11.19%; Y10, 13.44%). The wet season bacterioplankton communities at the Y10 site were significantly different from communities at other sites.

In addition to assigning phylum-level classifications to OTUs, they were also classified based on similarity searches against the database of freshwater bacteria (Newton et al., 2011; Figure 4B). The relative abundances of freshwater bacteria were significantly higher in the upper reaches than in the middle reaches (*p* = 0.028) in the wet season. In contrast, the relative abundances of freshwater bacteria were lower in the dry season upper reach communities compared to the other reaches communities. Non-freshwater bacterial abundances fluctuated among sampling sites and were similar to trends in bacterioplankton community diversity overall (Figure 2). In the dry season, non-freshwater bacterial abundances were more abundant at sites Y01 (29.41%) and Y02 (32.18%) than at other sites. Meanwhile, “others” (taxa with relative abundances <1%) exhibited highest relative abundance contributions in the upper reaches (Figure 4A). The proportion of non-freshwater bacteria was higher in the Yichun City (Y06, 23.05%; Y07, 22.38%) and Xinyu City (Y12, 27.04%) communities compared to other sites. In addition, Proteobacteria exhibited higher relative abundances at sites Y06, Y07, and Y12, with Epsilonproteobacteria being the dominant group (Figure 4A). Both the Shannon diversity index and Chao1 richness index were significantly and negatively correlated with freshwater bacterial abundances (*p* < 0.05), while proteobacterial abundances were negatively correlated with freshwater bacterial abundances (Supplementary Table 3).

### General Characteristics of Water Chemistry Along Yuan River

Water chemistry parameters, including pH, DO, DOC, NH_4_^+^-N, TP, NO_3_^–^-N, Cl^–^, SO_4_^2–^, and several heavy metals (Mn, Fe, Zn, and Pb), had spatial and temporal variability along Yuan River (Supplementary Figure 6 and Supplementary Table 4). DO levels were higher in the dry season than in the wet season, while the concentrations of Cl^–^ and SO_4_^2–^ were higher in the wet season than in the dry season. NH_4_^+^-N, TP, and NO_3_^–^-N reached significantly higher levels in the middle and lower reach samples compared to those in the upper reaches. Nutrient concentrations (DOC, NH_4_^+^-N, TP, and NO_3_^–^-N) were significantly and positively correlated with each other in the wet season samples. Cl^–^ was significantly and positively correlated with Fe, Zn, and Pb in wet season. DOC, Fe, Zn, and Pb exhibited a significantly higher value in dry season middle and lower reach samples compared to those in the upper reaches; DOC was significantly and positively correlated with NH_4_^+^-N; metals were significantly and positively correlated with each other. In addition, Fe concentrations were significantly and negatively correlated with Shannon and Chao1 indices with in the dry season, and the Shannon diversity index was significantly and negatively correlated with Zn concentrations that Mn were significantly and negatively correlated (Supplementary Figures 7, 8).

### Environmental Factors Related to Bacterioplankton Community Variation

**FIGURE 5:**
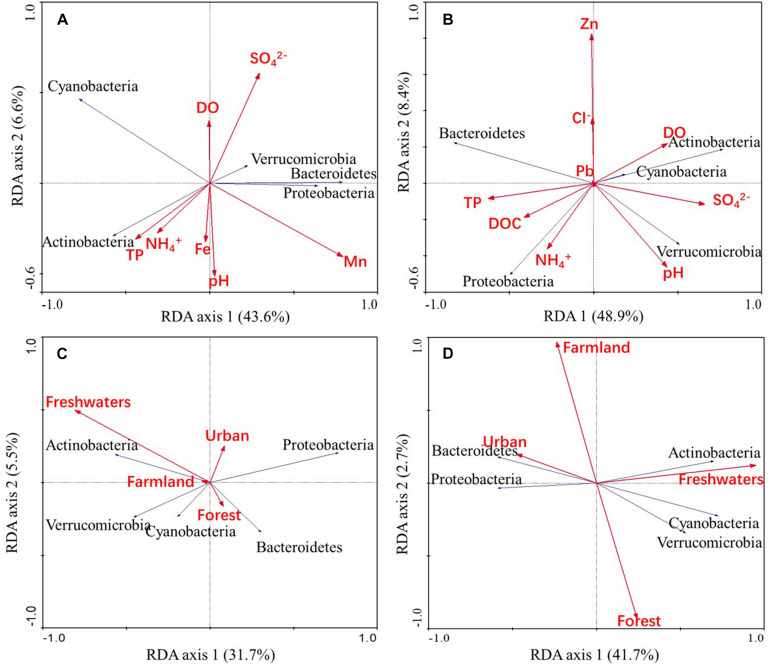
RDA ordinations showing bacterioplankton community variation in relation to water chemistry parameters and land use types. The abundances of predominant phyla in the bacterioplankton communities were related to water chemistry parameters in wet **(A)** and dry **(B)** season samples and were also related to land use patterns in wet **(C)** and dry **(D)** season samples.

Redundancy analysis (Figure 5) revealed that Mn (*p* = 0.014) and SO_4_^2–^ (*p* = 0.026) concentrations were statistically significant water chemistry parameters that were associated with bacterioplankton community composition (based on 499 Monte Carlo permutations). Freshwater area (*p* = 0.026) was a land use type that was significantly associated with bacterioplankton community composition (based on 499 Monte Carlo permutations). In addition, Actinobacterial abundances were positively correlated with freshwater area in the wet season, while Bacteroidetes abundances were negatively correlated with freshwater area in the dry season.

**FIGURE 6:**
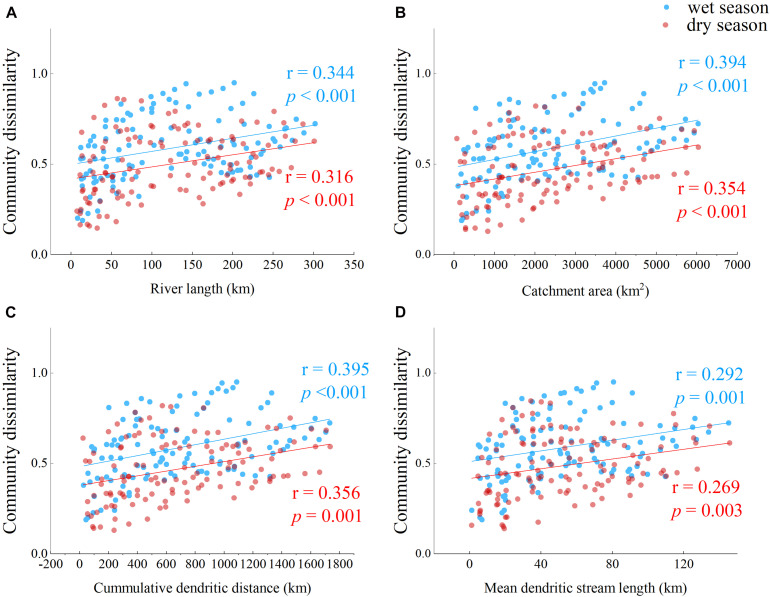
Bray–Curtis dissimilarity values of bacterioplankton communities in response to four geographic distance parameters. Relationships are shown between **(A)** river length (km), **(B)** catchment area (km^2^), **(C)** cumulative dendritic distance (km), and **(D)** mean dendritic stream length (km) with Bray–Curtis dissimilarity values for bacterioplankton communities. Pearson correlations (*r*) and probabilities (*p*) are shown to the right of each plot.

Geographic distances could also be important in structuring bacterioplankton assembly along the Yuan River and were thus evaluated (Figure 6). Cumulative dendritic distance appeared to describe bacterial community differences well (*r* = 0.395, *p* = 0.001 in the wet season; *r* = 0.356, *p* = 0.001 in the dry season). A slightly higher association was observed between the bacterial community similarity matrix and geographic distance in the wet season communities compared to those of the dry season.

Bioenv analyses (Table 1) indicated that the optimal subset of water chemistry parameters that best correlated to bacterioplankton OTU compositions included pH, SO_4_^2–^, Mn, and Fe (Bioenv correlation = 0.498) in the wet season; DO, DOC, SO_4_^2–^, Mn, and Fe levels comprised the optimal parameter subset associated with bacterioplankton composition (Bioenv correlation = 0.617) in the dry season. SO_4_^2–^, Mn, and Fe concentrations also exhibited the highest Spearman correlation with bacterioplankton communities in the wet and dry season. In terms of land use types, the optimal subset of parameters included forest and freshwater area (Bioenv correlation = 0.436 in the wet season; Bioenv correlation = 0.368 in the dry season). Catchment area, river length, and cumulative dendritic distance were selected by the Bioenv analysis as the combination that best represented the geographic distance parameters.

**TABLE 1:** Correlations between different combinations of environmental factors and bacterioplankton communities, as indicated by Bioenv analysis.

	**Combination in wet season**	**Correlation**	**Combination in dry season**	**Correlation**
Water chemistry	SO_4_^2–^	0.471	Mn	0.374
	pH + SO_4_^2–^	0.487	DO**+** Mn	0.532
	pH + SO_4_^2–^ + Fe	0.476	DOC + **SO_4_^2^**^–^**+** Mn	0.575
	**pH + SO_4_^2^**^–^**+ Mn + Fe**	**0.498**	DOC + **SO_4_^2^**^–^**+** Mn + Fe	0.603
	pH + DO + SO_4_^2–^ + Mn + Fe	0.481	**DO + DOC + SO_4_^2^**^–^**+ Mn + Fe**	**0.617**
	pH + DO + SO_4_^2–^ + Mn + Fe + Zn	0.438	pH + DO + DOC + SO_4_^2–^ + Mn + Fe	0.604
Land use	Freshwater	0.414	Freshwater	0.347
	**Forest + Freshwater**	**0.436**	**Forest + Freshwater**	**0.368**
	Forest + Freshwater + Urban	0.4188	Forest + Freshwaters + Urban	0.355
	Farmland + Forest + Freshwaters + Urban	0.353	Farmland + Forest + Freshwaters + Urban	0.340
Geographic distance	**Catchment area**	**0.437**	Cumulative dendritic distance	0.552
	Catchment area + Cumulative dendritic distance	0.436	**River length + Cumulative dendritic distance**	**0.561**
	River length + Catchment area + Cumulative dendritic distance	0.416	River length + Catchment area + Cumulative dendritic distance	0.560
	River length + Catchment area + Cumulative dendritic distance + Mean dendritic stream length	0.402	River length + Catchment area + Cumulative dendritic distance + Mean dendritic stream length	0.542

*Maximum values are shown in bold.*

**FIGURE 7:**
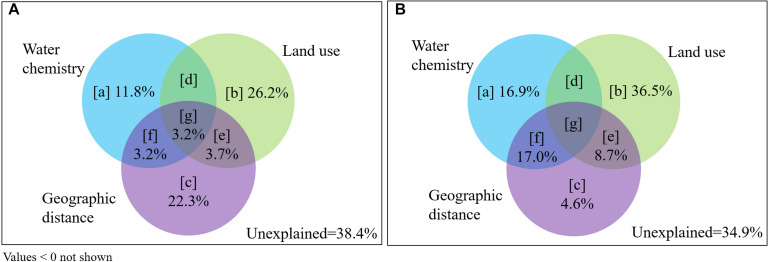
Partitioning of variation in bacterioplankton communities in the Yuan River. The effects on **(A)** wet season and **(B)** dry season communities were evaluated based on contributions from water chemistry, land use, and geographic distance parameters, following Supplementary Figure 2. Panels [a], [b], and [c] stand for the pure contribution of each explanatory matrix; panels [d], [f], and [e] stand for the joint contribution of two explanatory matrices; panel [g] stands for the joint contribution of three explanatory matrices. Unexplained: the variation not explained by the water chemistry, land use patterns or the geographic distance.

Variance partitioning analysis indicated that the variations in bacterioplankton communities explained by geographic distance [c + e + f + g] and land use [b + d + e + g] were higher than water chemistry [a + d + f + g] (Figure 7). The variation in communities explained by land use patterns alone [b] was higher than the other environmental factors (26.2% in wet season; 36.5% in dry season). There are share effect of above variables [g] (3.2%) in the wet season; only geographic distance contributed effect for water chemistry [f] (17%) in the dry season. Based on the results shown in Figure 7 and following the interpretations from Supplementary Figures 1, 3, the mass effect [b + c + e] (pure effect of land use patterns and geographic distance) contributed greater to variation in communities (52.2% in the wet season and 49.8% in the dry season) than species sorting [a + d + f + g] (effect of water chemistry) (9.4 and 15.3%, respectively).

## Discussion

Our study indicated that land use patterns and geographic distance surprisingly contributed more to bacterioplankton community variation than did water chemistry (Figure 7). These differences could be attributable to land use patterns and geographic distance affecting bacterioplankton communities to a greater extent due to the direct effects of allochthonous bacteria inputs and the spatially associated differences in water chemistry. The Pingxiang coal mines and Xinyu City exhibit high abundances of coal and iron storage and could contribute to significant allochthonous inputs into rivers (Wang et al., 2017) in addition to spatially associated geographic variation in communities. In the sites with abundant Urban areas (e.g., mines and cities), non-freshwater bacteria (e.g., Epsilonproteobacteria) were abundant in the water communities (Figure 4). The Epsilonproteobacteria were primarily represented by the genus *Arcobacter* (data not shown) that are commonly found in the digestive tracts of vertebrates (Hou et al., 2018). These non-freshwater populations could have been introduced via runoff and anthropogenic inputs (Zwart et al., 2002; Staley et al., 2013), reflecting the influence of mass effects (Supplementary Figure 1). This interpretation is supported by a stronger influence of mass effects on bacterial communities in the Le’an River compared to species sorting, which is likely due to mining activities. Furthermore, multiple water chemistry variables including SO_4_^2–^, Mn, and Fe concentrations significantly correlated with the composition of the bacterioplankton communities (Table 1 and Supplementary Figures 6–8). These results point to the influence of water chemistry on bacterioplankton communities due to the spatial distribution of land use patterns (Figure 7). SO_4_^2–^ concentrations in river waters are an important indicator of anthropogenic activities, especially in mining areas (Bhuiyan et al., 2010; Ako and Mfonka, 2018). Moreover, coal mining effluents, coal gangue dumping, and coal ash released by burning would lead to increased heavy metal pollution (Li and Sun, 2016; Shu et al., 2020).

Iron and Mn concentrations were significantly associated with bacterioplankton community variation (Table 1, Figure 5, and Supplementary Figures 7, 8). Fe and Mn (dissolved) could exert different pressures on the community, based on their bioavailability which is dependent on water pH or redox potential to changing the valence state of metals (Roane et al., 2015). The effects of these redox-sensitive heavy metals (Fe and Mn) on communities are particularly sensitive to seasonal variability (Supplementary Figure 6) due to fluctuating redox conditions (e.g., via DO; Supplementary Figure 5). Phytoplankton could influence the composition of bacterial communities via oxygen depletion (Fuhrman et al., 2015). Coclet et al. (2019) highlight both potential direct influences of metals on bacterioplankton and indirect forcing through biotic interactions. Effects of phytoplankton on bacterioplankton might have indirect consequences, depending on the possible interactions. While we neither evaluated the speciation of the main metals nor biotic interactions, future studies are needed to elucidate the contribution of bioavailability modulation and phytoplankton. Nevertheless, oxygen concentration has been demonstrated that the natural processes (season and biotic interactions) influence on metals and community, anthropogenic point sources are responsible for significant relationship between metal and diversity communities, especially in dry season (Supplementary Figures 7, 8). Previous studies have observed that DOC and pH are significant drivers of microbial community composition in rivers (Jones et al., 2009; Niño-García et al., 2016b; Pernthaler, 2017), although these parameters were not particularly important for explaining community compositions in the Yuan River. DOC can come from extracellular release and leachate from phytoplankton and macrophytes, and soil flow pathways, and may be enriched by domestic sewage or agricultural runoff (Allan and Castillo, 2007). An increase in stream water DOC during the wet season was expected due to shallow subsurface flow paths and flushing of soil DOC (Supplementary Figure 6). Allan and Castillo (2007) suggested that extracellular exuding organics are relatively small compared to allochthonous DOC sources, though this is an important local source. Anthropogenic influences can mask the seasonal pattern that organic matter exist from extracellular release and internal leaching in a river (Cole and Caraco, 2001). There is evidence that bacterioplankton communities in rivers adapt to changes in the concentration and composition of organic carbon (Kirchman et al., 2004; Judd et al., 2006), reflecting the influence of species sorting (Supplementary Figure 1). In addition, DOC was significantly correlated with NH_4_^+^-N contents and substantially changed at sites near Yichun city (Table 1, Supplementary Table 5, and Supplementary Figure 6). Samples in Yichun city made up more non-freshwater populations than other sample sites (Figure 4), reflecting the influence of mass effect (Supplementary Figure 1). These observations indicate that DOC was strongly associated with point pollution effects due to urbanization, and was not an overall influential factor. pH was also one of the water chemistry parameters chosen as a subset to best explain bacterioplankton community variation in the wet season, following SO_4_^2–^ (Table 1). Variations in both of these parameters are strongly associated with runoff from mines. Taken together, these results indicate that the input of allochthonous bacteria into the Yuan river increased during the wet season due to run off from cities and mining areas. Thus, the influence of allochthonous inputs from land use at a variety of river network locations cannot be ignored.

Low pH may also affect the availability of DOC to bacterioplankton because the cell membrane is less permeable to humic compounds at low pH (Edling and Tranvik, 1996). Cl^–^, which is also a good indicator of domestic wastewater (Lim et al., 2017), was significantly correlated with metals in the wet season (Supplementary Table 5), though not included in analysis in Bioenv. We acknowledge that it is not always possible to delineate between the mechanism behind water chemistry parameters heterogeneity and the bacterioplankton community could be simultaneously driven by natural and anthropogenic influence. It is not possible to discriminate single sources (e.g., domestic wastewater, industry, and agriculture) of the eutrophication underpinning the shifts in the microbial community, as Jeffries et al. (2016) also suggested. We are confident that our results provide a better understanding of anthropogenic influence on riverain ecosystems and the response of bacterioplankton communities.

Land use patterns alone contributed more to community variation than did other environmental factors. Forest land area and fresh water area were included as the best subset of land use variables to explain bacterioplankton community variation and could also potentially explain the input of allochthonous bacteria (Table 1). Forests are typical land use types in headwater streams where non-freshwater bacteria were abundant in this study, and especially in the dry season communities (Figures 1, 4). Hosen et al. (2017) did not document evidence of changes in water microbiome under the influences of forest land runoff, and indicators of bacterioplankton species or water chemistry were not found for forest land in our study. Instead, water temperature and river flow rate were important determinants with respect to the community structure (Chen et al., 2018), as also suggested in this current study. Headwater streams feature more advection of bacterial populations and proximity to groundwater supply due to closer to the bed of the stream and instream biota, as well as closer to adjacent stream banks, relative to lower reach sites. Groundwater is the major source of water supply in dry season, which makes the water contain more instream biota in the headwater streams. In general, dispersal typically increases local diversity (Cadotte, 2006), making river banks become sources of inputs, while stream water bodies become sinks (England and Rosemond, 2004; Lowe et al., 2006). In contrast, we found that forest area had higher bacterioplankton diversity and richness for headwater stream in the dry season (Figure 2). High influx rates creating the local environmental bacteria pool cannot maintain species diversity (Nilsson and Grelsson, 1995). Thus, we found that relative abundances of freshwater bacteria were significantly higher in the upper reaches in the wet season (Figure 4). Vilmi et al. (2016) suggested that local diversity reaches its maximum at an intermediate level of dispersal. These observations suggest that mass effects more strongly influence bacterial communities in the upper Yuan River than species sorting.

In specific freshwater conditions like that are present in the region of lake/reservoir, water flow rate slows down, residence time increases, and DO concentrations decrease, resulting in less bacterioplankton dispersal or dilution due to habitat filtering (i.e., species sorting; Supplementary Figure 1). After bacterioplankton communities transit the lake/reservoir, these conditions promote the growth of anaerobic bacteria (e.g., Planctomycetes and Parcubacteria) and K-strategist bacteria (e.g., Actinobacteria) (Whitman and Bergey’s Manual Trust, 2015), while inhibiting the growth of R-strategist bacteria (e.g., Bacteroidetes) (Adams et al., 2014). Lindström et al. (2006) suggested that bacterioplankton communities in lake with residence times <100 days exhibit signatures of mass effects, as reflected by similarities of bacterial communities at inlet and outlet like the previous work (Adams et al., 2014). The residence time of the Jiangkou reservoir is less than 100 days, and thus a mass effect still likely exists.

Water residence time was described by the mean dendritic stream length metric in the Danube River (Savio et al., 2015) and was observed to be related to species sorting (i.e., via predation or competition; Supplementary Figure 1; Wang et al., 2019). Similarly, Liu et al. (2018) also observed that mean dendritic stream length better described the spatial similarity of bacterial communities in the Yangtze River rather than cumulative dendritic distance. In the present study, cumulative dendritic distance best explained bacterioplankton community variation among the geographic distance parameters that were analyzed (Figure 6). The Yuan River (length = 297 km) has a shorter water residence time than the Yangtze River (6,300 km) and Danube River (2,850 km), and thus the cumulative dendritic distance may better describe water residence times in smaller scale catchments. This observation is consistent with results for a study of the River Thames (346 km), which provided evidence for a clear relationship between bacterial community composition and cumulative dendritic distance (Read et al., 2015). Liu et al. (2018) hypothesized that cumulative dendritic distance reflects the river network density and is related to species sorting and mass effects (i.e., due to predation, growth competition, and allochthonous inputs; Supplementary Figure 1). In our study, catchment area became the single best geographic distance parameter to explain community dissimilarity in wet season samples (Table 1), reflecting both the local environmental conditions (e.g., through species sorting) and the unique locations of the river network (e.g., evidence for mass effects). Altermatt et al. (2013) observed that species richness generally increased with increasing catchment area. In the dry season, river length and cumulative dendritic distance were the geographic distance parameter subset that best explained bacterial community dissimilarities (Table 1). River length reflects anthropogenic effects and is related to mass effects (e.g., due to allochthonous inputs; Figure 1; Wang et al., 2019). Thus, our results suggest that mass effect was the dominant mechanism shaping the bacterial communities in the Yuan River.

## Data Availability Statement

The original contributions presented in the study are publicly available. These data can be found in the NCBI Sequence Read Archive database (accession number: SRP194014).

## Author Contributions

WP designed the study. WP, JZ, MD, MN, and GH collected the samples. JZ analyzed the genetic data. JZ, MD, MN, and GH analyzed the geographic data. WP and JZ wrote the manuscript with contributions from all co-authors. All authors approved the final manuscript for publication.

## Conflict of Interest

The authors declare that the research was conducted in the absence of any commercial or financial relationships that could be construed as a potential conflict of interest.

## Footnotes

1 http://www.gscloud.cn

2 http://qiime.org/scripts/assign_taxonomy.html

3 http://drive5.com/uparse/

4 http://sourceforge.net/projects/rdp-classifier/

5 http://www.arb-silva.de

6 https://www.mothur.org/wiki/Download_mothur
