# Supplementary material for: Effect of Water Chemistry, Land Use Patterns, and Geographic Distances on the Spatial Distribution of Bacterioplankton Communities in an Anthropogenically Disturbed Riverine Ecosystem
Source: Front Microbiol. 2021 May 6;12:633993. doi: 10.3389/fmicb.2021.633993 (PMC8138559; doi:10.3389/fmicb.2021.633993)
Supplement: Supplementary file 1 [file Data_Sheet_1.PDF]

Supplementary Material

1 Supplementary Figures and Tables

1.1 Supplementary Figures

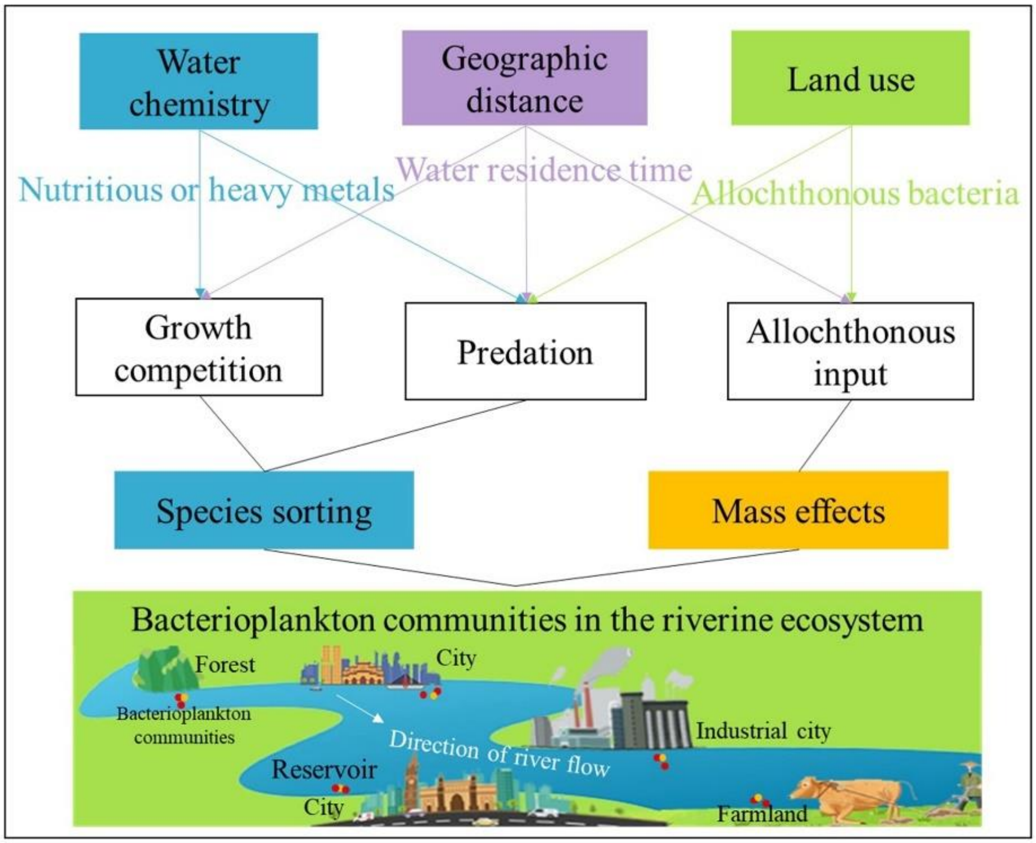

Supplementary Figure 1. Schematic representation showing the upstream and downstream influences of water chemistry, geographic distance, and land use on bacterioplankton communities in the Yuan River. The shown factors affect the spatial distribution of bacterioplankton communities by regulating environmental interactions including predation, growth competition, and allochthonous bacterial inputs. Predation and growth competition are associated with the species sorting determinant mechanism of community assembly, while allochthonous input is related to the mass effect determinant mechanism.

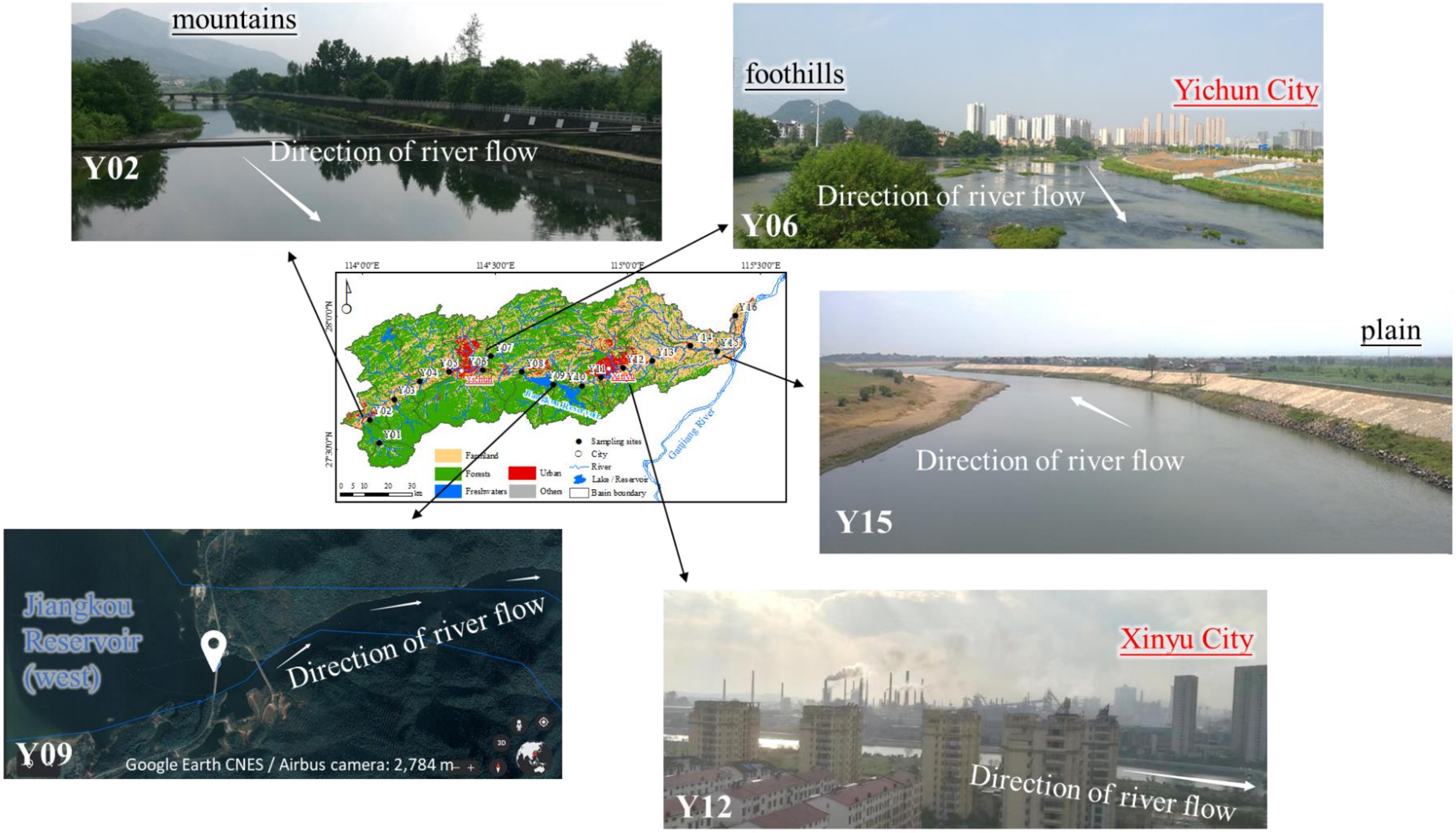

Supplementary Figure 2. Detailed map of the sampling location, with the various landforms. The Yuan River passes through mountains, foothills, and plains. Sampling sites were chosen evenly to represent a high diversity in environmental gradients, e.g., variety in land use. GPS coordinates of the sampling sites can be found in the Supplementary table 1.

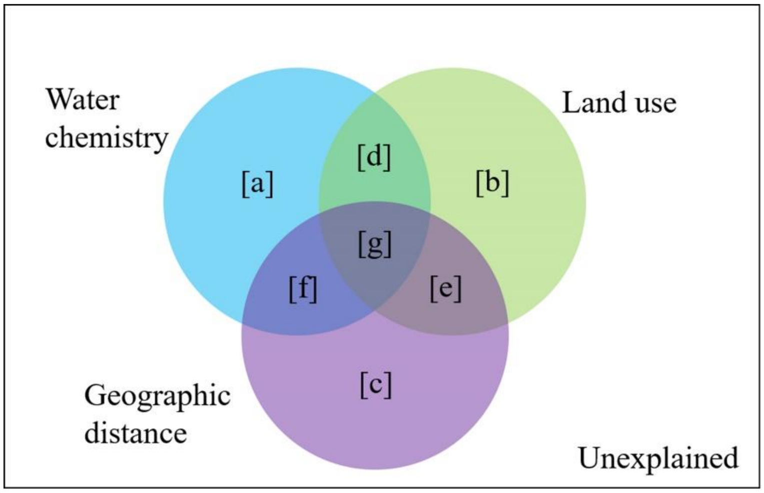

Supplementary Figure 3. A schematic of the variance partitioning analysis (VPA) modeling that was used to quantify the relative contributions of water chemistry, land use, and geographic distance parameters to bacterioplankton community variation. Water chemistry: variation explained by water chemistry parameters. Land use: the variation explained by land use parameters. Geographic distance: the variation explained by geographic distance parameters. Unexplained: variation not explained by water chemistry, land use, or geographic distance parameters.

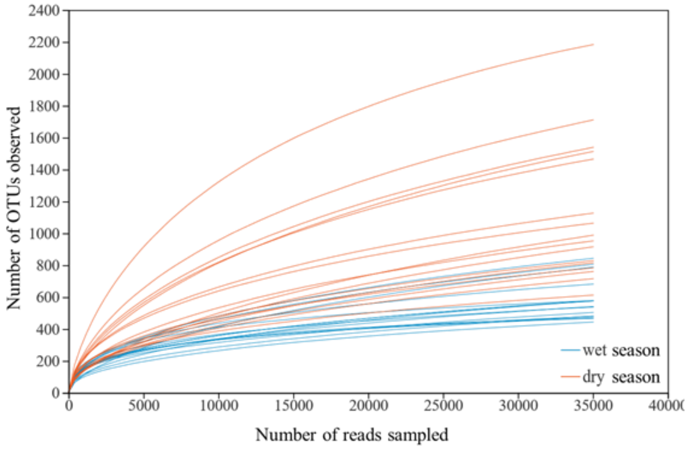

Supplementary Figure 4. Rarefaction curves of operational taxonomic units (OTUs) at the 97% nucleotide sequence similarity level.

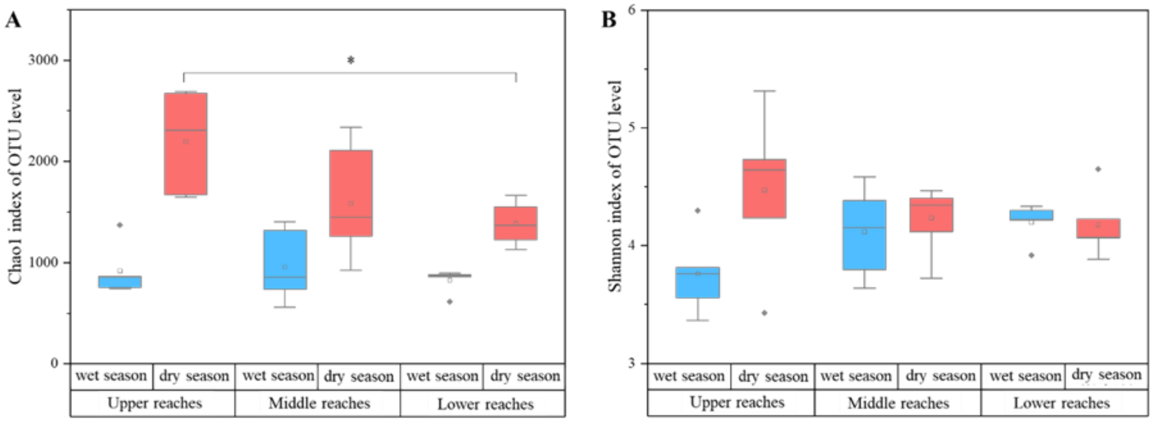

Supplementary Figure 5. Comparison of Chao1 richness and Shannon diversity indices of communities from along the upper and downstream reaches of the Yuan River. Boxes show means ± SE, while whiskers show means ± SD. Wet season distributions are in blue and dry season distributions in red. * indicates a statistically significant difference at $p < 0.05$ (one-way ANOVA).

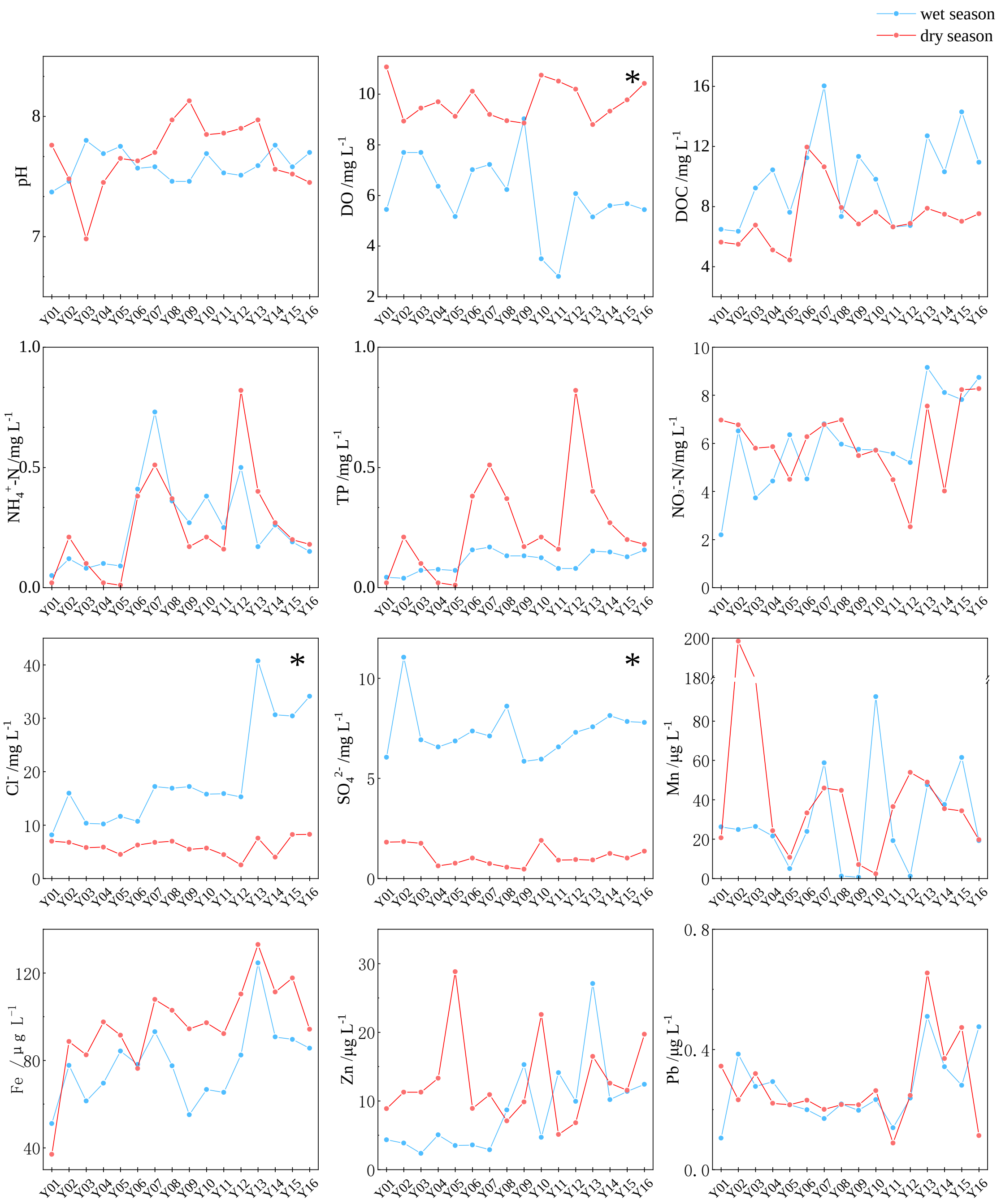

Note: * indicates a statistically significant difference across seasons (p < 0.05, t-test).

Supplementary Figure 6. Water chemistry parameter measurements arranged from upstream to downstream sites.

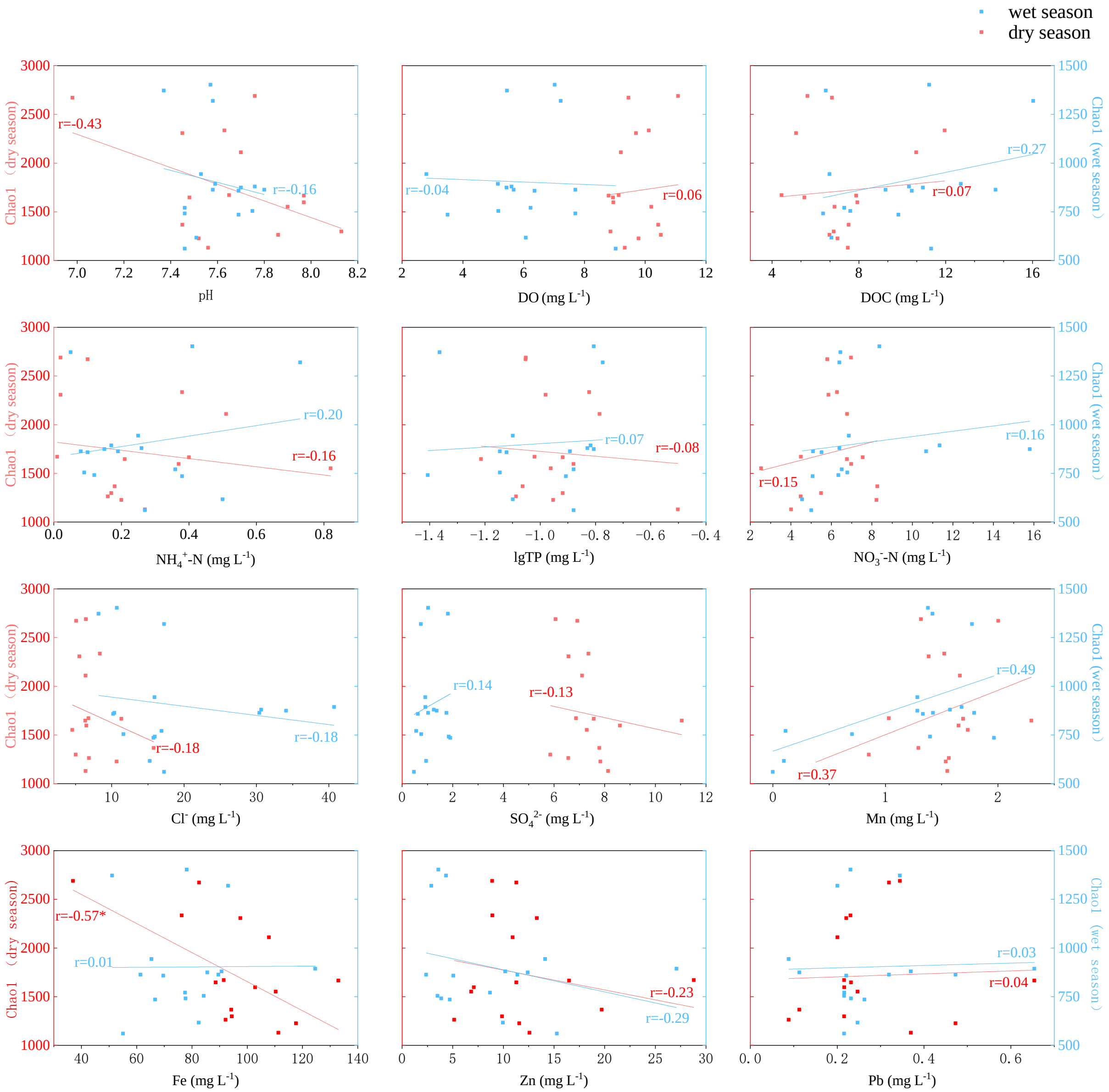

Note: * indicates statistically significant correlations ($p < 0.05$).

Supplementary Figure 7. Scatter plot of correlations between Chao1 index values and water chemistry parameters.

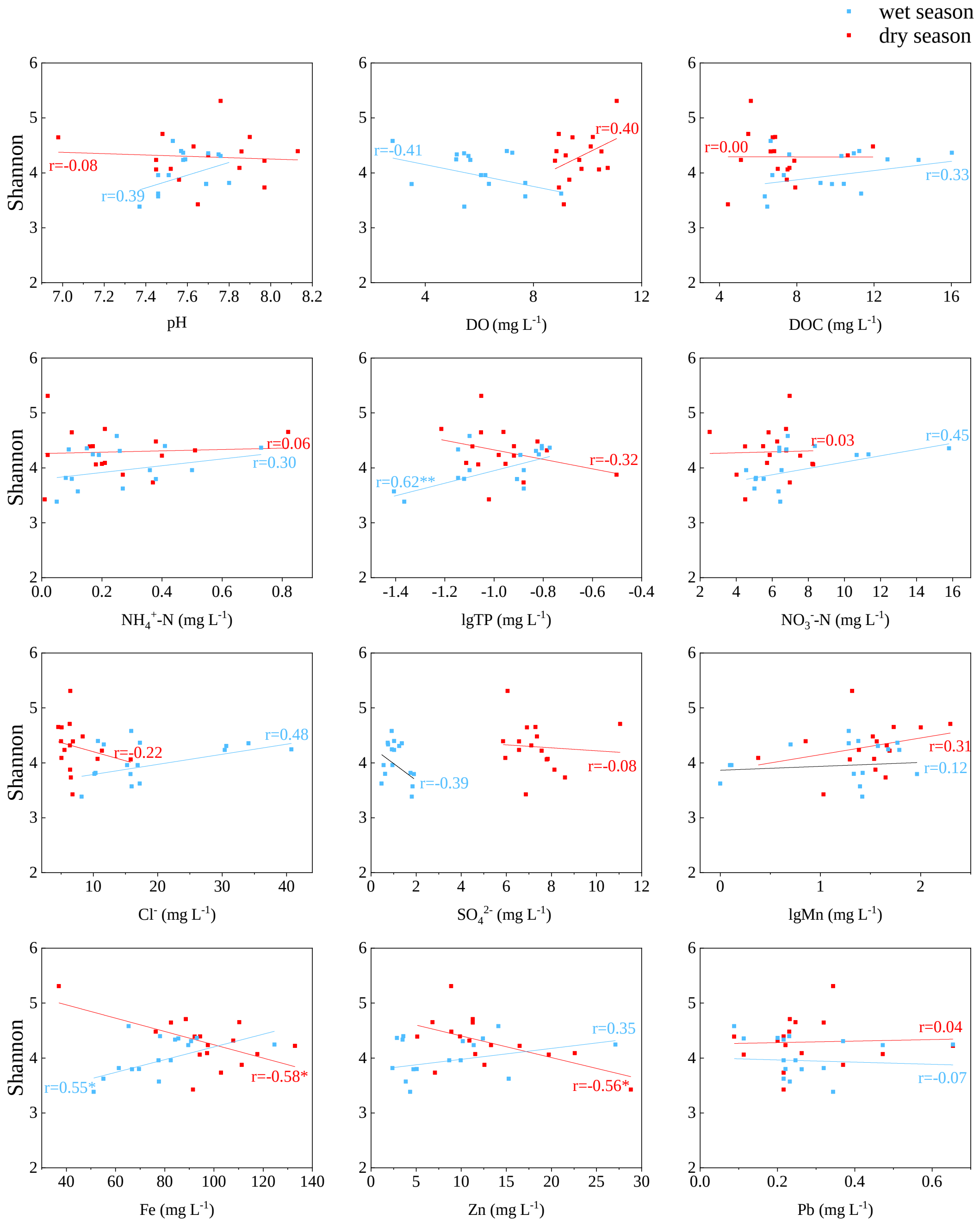

Note: * indicates statistically significant correlations ($p < 0.05$).

Supplementary Figure 8. Scatter plot of correlation between Chao1 richness index values and water chemistry parameters.

1.2 Supplementary Tables

Supplementary Table 1. Locations information and parameters of land use pattern and geographic distance for 16 sampling sites in the Yuan River basin.

	Site	Lat. (°N)	Lon. (°N)	Land use (%)					Geographic distance			
				Farmland	Forest	Freshwaters	Urban	Others	River length (km)	Catchment area (km^2)	Cumulative dendritic distance (km)	Mean dendritic stream length (km)
**Upper reaches**	Y01	27.52	114.07	11.12	84.41	0.11	1.17	3.18	8.41	137.72	36.88	11.36
	Y02	27.61	114.03	13.80	80.89	2.81	1.26	1.23	22.43	235.89	64.36	22.56
	Y03	27.69	114.12	40.18	45.52	0.87	11.83	1.6	36.87	420.46	109.56	29.45
	Y04	27.75	114.22	22.58	72.14	1.34	2.04	1.9	51.75	1142.18	328.00	40.76
	Y05	27.79	114.33	34.95	58.95	0.79	2.96	2.36	64.92	1621.95	451.07	46.79
**Middle reaches**	Y06	27.80	114.46	18.34	69.44	0.89	8.82	2.5	80.62	2,270.11	638.76	56.81
	Y07	27.85	114.49	33.15	38.04	1.50	23.85	3.45	88.75	2,491.74	696.98	62.63
	Y08	27.79	114.60	32.80	60.26	1.55	4.05	1.34	110.41	3,324.81	922.56	67.65
	Y09	27.74	114.72	15.97	70.19	8.13	4.68	1.09	150.75	3,617.69	1,022.74	82.65
	Y10	27.74	114.83	10.00	78.85	8.94	1.23	0.98	210.36	3,861.08	1,125.31	92.02
	Y11	27.77	114.90	35.47	50.02	2.04	10.11	2.35	222.52	4,203.78	1,209.48	97.81
**Lower reaches**	Y12	27.80	114.98	35.18	44.69	2.03	15.90	2.2	234.62	4,829.92	1,366.32	102.60
	Y13	27.83	115.09	44.67	33.43	2.72	16.50	2.68	253.63	5,001.86	1,425.33	118.25
	Y14	27.89	115.24	72.25	11.75	3.31	8.20	4.49	274.70	5,313.52	1,520.08	136.48
	Y15	27.87	115.34	63.75	21.26	3.80	6.51	4.69	286.84	5,859.60	1,667.25	137.66
	Y16	28.00	115.41	62.26	22.60	3.33	5.21	3.59	310.46	6,169.75	1,768.39	156.58

Supplementary Table 2. Numbers of OTUs, sequence reads, and taxonomic groups for bacterioplankton communities in the Yuan River.

	OTUs	Reads	Phyla	Classes	Orders	Families	Genera	Species
Wet season	2,074	892,792	41	90	173	314	607	1,006
Dry season	3,634	933,377	46	116	209	378	816	1,556

Supplementary Table 3. Relative abundances of the five most dominant phyla in the Yuan River ecosystem samples. Samples are grouped following Figure 5.

	Site	Proteobacteria	Actinobacteria	Bacteroidetes	Cyanobacteria	Verrucomicrobia
**Wet season**	Group 1	45.23±13.24%[a]	16.38±4.11%[b]	31.56±5.69%[a]	0.52±0.13%[b]	5.10±4.35%
	Group 2	38.21±13.72%[a]	35.27±9.26%[a]	17.01±4.25%[b]	4.75±5.87%[b]	2.49±1.61%
	Group 3	18.31±5.72%[b]	48.02±16.69%[a]	5.59±1.23%[c]	20.67±12.44%[a]	2.59±1.72%
	Group 4	34.96±5.62%[a]	35.51±7.80%[a]	18.74±4.54%[b]	3.31±4.30%[b]	4.09±3.69%
	Y10	23.81%	50.36%	7.76%	0.45%	6.82%
**Dry season**	Y01	34.62%	32.52%	16.84%	2.01%	1.85%
	Y02	32.64%	34.38%	7.37%	1.12%	4.37%
	Group 1	35.58±4.49%[a]	15.98±6.93%[c]	39.97±8.91%[a]	0.46±0.53%[c]	0.41±0.21%[b]
	Group 2	27.08±4.55%[b]	46.79±8.00%[a]	14.96±4.72%[c]	5.03±4.62%[a]	2.59±1.64%[a]
	Group 3	34.51±3.51%[a]	31.84±5.29%[b]	28.56±3.46%[b]	0.81±0.41%[b]	0.98±0.47%[b]

Note: Lowercase letters indicate statistically significant differences ($p < 0.05$, ANOVA test, Tukey's HSD).

For site names, see **Figure 3**.

Supplementary Table 4. Pearson correlations between the proportion of freshwater bacteria and the relative abundances of dominant bacterial phyla/alpha-diversity index values.

	Proportion of freshwater bacteria in the wet season communities	Proportion of freshwater bacteria in the dry season communities
**Proteobacteria**	0.56*	-0.24
**Actinobacteria**	-0.33	0.08
**Bacteroidetes**	0.76**	0.43
**Cyanobacteria**	-0.89**	-0.16
**Verrucomicrobia**	0.04	-0.50*
**Shannon index**	-0.08	-0.85**
**Chao1 index**	0.44	-0.56*

*: Correlation is statistically significant at the $p < 0.05$ level; **: correlation is statistically significant at the $p < 0.01$ level

Supplementary Table 5. Pearson's correlation values for water chemistry parameter comparisons.

	**pH**	**DO**	**DOC**	**NH_4^+-N**	**Lg(TP)**	**NO_3^--N**	**Cl^-**	**SO_4^{2-}**	**Lg(Mn)**	**Fe**	**Zn**	**Pb**
**pH**	1											
**DO**	-0.16/-0.04	1										
**DOC**	0.25/0.13	0.22/0.01	1									
**NH_4^+-N**	-0.16/0.37	0.08/-0.10	0.38/**0.54***	1								
**Lg(TP)**	0.27/0.12	-0.02/-0.30	**0.73****/0.48	**0.53***/0.34	1							
**NO_3^--N**	0.27/-0.17	-0.15/-0.06	0.47/0.21	0.11/-0.27	**0.58***/-0.20	1						
**Cl^-**	0.15/-0.11	-0.18/0.05	0.47/0.22	0.08/-0.08	**0.60***/0.01	**0.94**/0.69****	1					
**SO_4^{2-}**	-0.13/-0.46	0.24/0.46	-0.15/-0.15	-0.07/-0.24	-0.16/-0.41	0.41/0.15	0.32/-0.02	1				
**Lg(Mn)**	0.36/-0.41	-0.37/-0.35	0.40/0.06	-0.08/0.29	0.07/0.03	0.18/0.09	0.18/0.09	0.06/0.08	1			
**Fe**	0.23/0.19	-0.14/**-0.52***	0.48/-0.16	0.20/0.48	0.49/0.34	**0.80****/-0.03	**0.73****/0.22	0.39/-0.45	0.27/0.10	1		
**Zn**	-0.15/-0.14	-0.21/-0.06	0.21/-0.27	-0.10/-0.37	0.39/-0.18	**0.60***/0.09	**0.73****/0.24	-0.03/0.15	-0.11/**-0.52***	**0.52***/0.13	1	
**Pb**	0.36/0.03	0.03/-0.26	0.22/0.01	-0.31/0.09	0.22/0.24	**0.72****/0.27	**0.71****/0.24	**0.50***/0.11	0.24/0.15	**0.66****/0.37	**0.50***/0.08	1

*: Correlation is statistically significant at the 0.05 level; **: correlation is statistically significant at the 0.01 level.

Note: First value shows the wet season value, and the second shows the dry season value
